# Effects of environmental noise on quantum charge diffusion in DNA sequences

**DOI:** 10.1038/s41598-025-02819-w

**Published:** 2025-05-20

**Authors:** Mirko Rossini, Ole Ammerpohl, Reiner Siebert, Joachim Ankerhold

**Affiliations:** 1 Ulm University, Institute for Complex Quantum Systems, 89069 Ulm, Germany; 2 Center for Integrated Quantum Science and Technology (IQST), Ulm, Germany; 3 Ulm University and Ulm University Medical Center, Institute of Human Genetics, 89069 Ulm, Germany

**Keywords:** DNA, Quantum effects, Charge diffusion, Tight-binding model, Decoherence, DNA, Computational biophysics, DNA, Biological physics, Electronic properties and materials, Quantum simulation

## Abstract

Charge fluctuations along stacked nucleobases in the DNA double helix play a key role in processes such as DNA repair and replication. While classical charge transfer mechanisms between adjacent bases due to energetic excitations are well established, quantum effects can also contribute significantly. Specifically, the overlap of $\pi$-orbitals in well-stacked nucleobases can enable charge delocalization along the DNA double-strand. However, the cellular environment, including water, surrounding molecules, and thermal noise, is thought to induce rapid decoherence, limiting quantum-enhanced charge transport under physiological conditions. To explore charge mobility in such noisy environments, we model quantum diffusion in DNA-inspired two-dimensional tight-binding lattices, considering intrinsic and environmental fluctuations and revealing, via atomistic parametrization, a complex network of charge transport pathways. Our results show that long-range quantum phenomena depend on the carrier type (electrons or holes), base sequence, and noise/disorder characteristics. Notably, spatially correlated low-frequency fluctuations can sustain coherent charge transfer across several bases, whereas moderate vibrational noise can enhance rather than suppress quantum coherence by facilitating tunneling effects. These findings suggest that even under physiological conditions, the DNA structure can support non-classical charge dynamics, offering insights into its potential role in bioelectronic processes and inspiring future models of quantum transport in biological systems.

## Introduction

The fluctuation of charges through DNA is a basic physicochemical phenomenon that has recently been shown to be involved in a number of key cellular processes in various living beings, including DNA replication and repair^1–6^. For example, charge transfer through DNA guides the binding of several sulfur-iron-containing enzymes to DNA, including the DNA repair enzymes of the MutY family in various species. As charge transfer through DNA requires proper stacking of the nucleobases, any DNA mismatch interferes with the charge transfer ability of DNA, which allows rapid scanning of DNA integrity by MutY and its homologs^2,3^.

From a physics perspective, the description of charge fluctuations through a chain of well-stacked DNA bases varies significantly depending on the different types of charge carriers and different kinds of fluctuations allowed by the system. Types of charge carriers include e.g. an electron (negative charge), a hole (a positive charge, caused by the “absence” of an electron), or an exciton (a bound state of an electron and a positively charged hole attracted to each other by the Coulomb force)^7^. Charge fluctuations supported by the DNA double-chain can originate from both classical physics principles or quantum physics principles, for example relying on the overlap of pi-orbitals of well-stacked nucleobases to move along the chain^8–10^. This last quantum-mediated mechanism for charge fluctuation along the chain is often of interest for shorter strands of DNA compared with charge-hopping mechanisms, but it enables novel channels for charge fluctuations along the chain resulting from quantum effects, such as quantum tunneling. Quantum tunneling allows a charge to move from one base to another, separated by an energetically inaccessible obstacle (e.g. another DNA base), by “tunneling” through it, even when such a process would be impossible according to the laws of classical physics. In this article, we focus mostly on understanding the quantum mechanical delocalization of electrons (negative charges) in such short stretches of DNA.

For an electron to diffuse along the DNA chain via quantum physical enabled paths, the electron requires “quantum coherence”, i.e. the ability of different quantum states to interfere with each other^11^. However, this coherence is highly sensitive to environmental interactions. Each time the quantum system engages with external factors, a process called decoherence or dissipation occurs, disrupting the coherence and hindering the electron’s movement^11^. With respect to DNA, its core nucleobase stack, which is essential for charge delocalization, is coated with a negatively charged sugar-phosphate backbone and, in living contexts, embedded in aqueous media. This biological surrounding of the nucleobases, which can be regarded as environmental or thermal noise, has been frequently postulated to interact with quantum mechanical processes in DNA leading to decoherence, i.e. the loss of coherence, and may, thus, mask the nucleotide-based quantum charge transfer. Thus, investigating the effects of the environmental bath surrounding the nucleobases on quantum mechanical charge diffusion in DNA is essential. In addition, the fact that the interaction of the surrounding bath with the DNA, in principle, also has the potential to determine epigenetic effects(^8^ and references therein) was not yet fully considered. Notably, epigenetic processes such as the enzymatic methylation of DNA or the binding of some transcription factors also alter charge diffusion processes in DNA^12–15^.

In the present study, we explored the role of the environment in the quantum physical properties of DNA by modeling electron (negative charge) diffusion in double-stranded DNA while taking into account different sources of thermal noise and disorder. To this end, we make use of a simplistic coarse-graining mathematical model known as tight-binding lattice model. Such double-stranded tight-binding models have been used previously to study the quantum dynamics of electronic excitations along nucleic acid sequences^16–19^. However, most previous studies that used this approach did not account for the impact of disorder, noise, and fluctuations induced by external (water, solvent, presence of counterions, substrate, or phonons), and internal (nuclei, base-pair sequence, or geometry) factors that are omnipresent in an ambient living environment. To address the intricate interplay of metallic-like behavior (coherent charge transfer) with disorder and noise induced by surrounding degrees of freedom, our model considers shorter DNA oligomers, where quantum coherence is expected to survive. Most importantly, we parametrize the tight-binding structure with values originating from atomistic simulations designed to resemble the DNA chemical properties and structure^20^.

Our findings show that the interplay between the quantum coherence survival of a charge diffusing along the DNA double-strand and its interaction with the external environment can generate non-classical behavior of the charge on the chain. By studying DNA chain models of different short strands interacting with a thermal bath (higher frequency noise), we are able to show that, depending on the specific sequence under analysis, the suppression of quantum coherence is not necessarily fast enough to prevent quantum phenomena such as quantum tunneling. In addition, our analyses of the interaction of the system with structural disorder (associated with vibrations and lower frequency noise) suggest that, counter-intuitively, small amounts of vibration could even support the coherent dynamics of a particle along the DNA chain, rather than suppressing it.

**Fig. 1:**
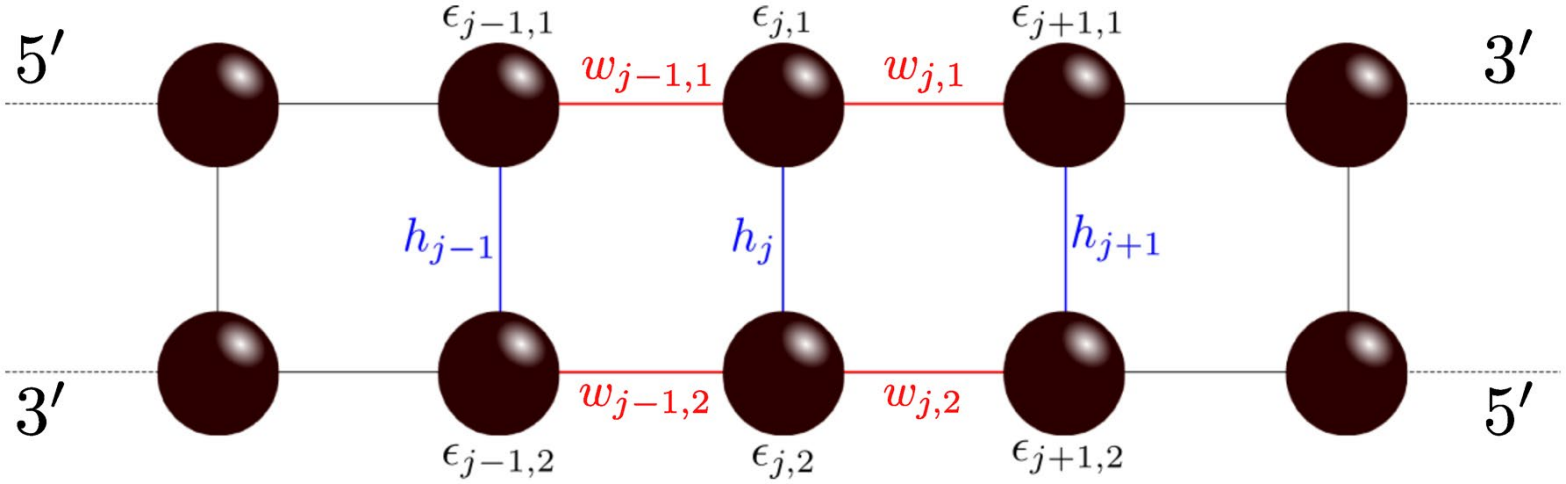
TB DNA structure: Schematic representation of the tight-binding *Ladder Model* (LM) used to mimic the structure of DNA.

## Methods

### A mathematical model for charge diffusion in the DNA structure

We consider a well-established^21^ model of the geometry and structure of DNA in the form of a double-strand tight-binding ladder, called *Ladder Model* (LM) (see Fig. 1) which has been introduced previously^19,22^. The LM is a minimal model that can address the influence of both base pairing and base stacking contributions on the energetics of the system. It consists of two tight-binding chains, denoted by the index $l\in L, \ \ L=\{1,2\}$, where individual sites on each chain *l* (nucleotides) are denoted by $1\le j\le n$ with localized electronic (hole) states $|l, j\rangle$. The LM is parametrized by on-site energies of each base, $\epsilon _{j,l}$, intra-strand hopping integrals between successive bases, $w_{j,l}$, and inter-strand hopping integrals, $h_j$ (see Fig. 1 for a schematic representation). Here, the HOMO (Highest Occupied Molecular Orbital) represents the highest energy level that naturally hosts electrons in a molecule, while the LUMO (Lowest Unoccupied Molecular Orbital) is the lowest energy level available for accepting electrons; together, these states play a key role in determining how molecules interact and transfer charge, as they define the molecule’s ability to donate or accept electrons. Accordingly, the Hamiltonian reads

1 $$\begin{aligned} H_{LM}=H_\textrm{loc}+H_\textrm{tun} \end{aligned}$$

consisting of two parts: A local part describing on-site energies, $H_\textrm{loc}$, and a non-local (tunneling) part describing interactions among different sites of the double chain, $H_\textrm{tun}$, i.e.

2 $$\begin{aligned} H_\textrm{loc} = \sum _{l\in L}\sum _{j=1}^N \epsilon _{j,l}|j,l\rangle \mathinner {\langle {j,l}|} \end{aligned}$$

and

3 $$\begin{aligned} H_\textrm{tun} = \sum _{l\in L}\sum _{j=1}^{N-1} w_{j,l}|j,l\rangle \mathinner {\langle {j+1,l}|} + h.c. \nonumber \\ + \sum _{j=1}^N h_{j}|j,1\rangle \mathinner {\langle {j,2}|} + h.c. \end{aligned}$$

The LM allows for a straightforward solution in case of infinite chains and site-independent parameters $\epsilon$, *w*, *h* throughout^19^. Even though this is a rather simplistic limit, it is nevertheless intriguing to briefly recall the main results. Assuming all parameters to be real-valued and positive and putting the lattice constant $a=1$, one finds energy bands

4 $$\begin{aligned} E_{\pm }(k) = \epsilon + 2w \cos (k) \pm h\, , \ \ \ \ \ -\pi \le k \le \pi , \end{aligned}$$

with width *w* and spacing *h*. They correspond to delocalized Bloch waves with quasi-momentum $-\pi \le k\le \pi$.

For chains of finite length *N* the quasi-momentum takes discrete values, i.e.,

$$\begin{aligned} k_n = \frac{2\pi }{N} \left( n-\frac{N}{2}\right) \, ,\ \ \ \ \ n = 0,...,N \end{aligned}$$

and the expression in Eq. (4) remains valid upon substituting $k\rightarrow k_n$. Accordingly, molecular energy bands related to HOMO and LUMO cover a range from $E_\textrm{min}=E_-(\pi )$ to $E_\textrm{max}=E_+(0)$ and support coherent long-range charge transfer through the chain. For chains of growing length the expected density of states with pronounced maxima emerges. To illustrate the situation, we display an example with parameters $\epsilon = 0$, $w = 1$, and $h = 0.5$ and varying length *N* in the Supplementary Materials - Fig. S1.

### Modeling the interaction between system and environment

As outlined in the introduction, realistic modeling has to include environmental degrees of freedom consisting e.g. of local vibrational modes as well as fluctuating modes of lower frequency stemming from the DNA backbone, which are not taken into account by the simplified LM model outlined above. Since a complete atomistic description is not feasible, one has to resort to an effective description in terms of various kinds of noise models. In the sequel, we will start with broadband dynamical noise sources accounting for different possible sources of local dissipation, describing the local interaction of each DNA base with the environment, and then proceed with various kinds of non-local sources for decoherence.

#### Effect of broadband noise sources on local dissipation at single bases

For broadband dynamical quantum noise, we follow the standard approach to describe the impact of thermal environments on the quantum dynamics within the tight binding structure in case of weak interaction^11^. The reasoning here is that charge transfer through DNA has been shown to be relatively long-ranged and relatively fast compared to classical processes, thus indicating relatively long-range coherence. From now on, we will consider the dynamics of a single charge along the chain.

Due to weak coupling a perturbative treatment applies to derive an effective equation of motion for the reduced density operator $\rho (t)=\textrm{tr}_R\{W(t)\}$, where all environmental degrees of freedom are traced out from the total density operator *W*. More specifically, the total compound is described by a Hamiltonian of the form $H_{tot} = H_{S} + H_R + V$ with $H_S=H_{LM}$, $H_R$ modeling the reservoir modes, and *V* capturing a bilinear coupling between LM and reservoir, i.e., $V= \sum _p A_p \, X_p$ with hermitian system operators $A_p$ and collective reservoir operators $X_p$. We note that the DNA-reservoir coupling can be very different in nature: For example, a uniform coupling, where all sites interact with the same reservoir, can be realized by setting $X\equiv X_i$ for all sites $i=1,\ldots , N$ with non-local system couplings $A_\nu$, while in a fully local coupling scheme site-dependent operators $X_i\ne X_j$ ($i\ne j$) appear with local operators $A_i$, while. All kinds of intermediate cases are possible as well, of course, but for the sake of clarity we will consider these limiting cases only.

Under the assumption of factorizing initial states $W(t) = \rho (t) \otimes \rho _R(t)$ with reservoir equilibrium at inverse temperature $\beta =1/k_\textrm{B} T$, i.e. $\rho _R=\exp (-\beta H_R)/Z_R$ [$Z_R=\textrm{Tr}[\exp (-\beta H_R)]$], and a time scale separation between the relaxation of the reduced system and the decay of equilibrium reservoir-reservoir correlations $\langle X_\nu (t)X_\mu (0)\rangle _\beta$, a Born-Markov treatment is valid. Together with a rotating wave approximation, this leads to

5 $$\begin{aligned} \dot{\rho }(t) = -i\left[ H_S,\rho (t)\right] + \mathcal {L}[\rho (t)] \end{aligned}$$

with Lindblad superoperators of the form

6 $$\begin{aligned} \mathcal {L}[\rho ] & = \sum _{\alpha , \beta }\sum _{\nu } \gamma _\nu (\omega _{\alpha \beta })\big [ A_\nu (\omega _{\alpha \beta })\rho (t)A_\nu ^{\dagger }(\omega _{\alpha \beta }) \\ & \quad - \frac{1}{2} \{ A_\nu (\omega _{\alpha \beta })A^{\dagger }_\nu (\omega _{\alpha \beta }),\rho (t) \} \big ]. \end{aligned}$$

Here, the operators $A_\nu$ and $A^{\dagger }_\nu$, inducing transitions between eigenstates of the system Hamiltonian $H_S|\alpha \rangle = E_\alpha |\alpha \rangle$, are represented in the form

7 $$\begin{aligned} A_\nu (\omega _{\alpha \beta }) = |\alpha \rangle \mathinner {\langle {\alpha }|}A_\nu |\beta \rangle \mathinner {\langle {\beta }|} \end{aligned}$$

with transition frequencies $\omega _{\alpha \beta } = E_\alpha - E_\beta$. The interaction with the reservoirs is characterized by transition rates

8 $$\begin{aligned} \gamma _\nu (\omega _{\alpha \beta }) = \int _{-\infty }^{\infty } dt\ \textrm{e}^{i\omega _{\alpha \beta }t} \langle X_\nu (t) X_\nu (0) \rangle _\beta \, , \end{aligned}$$

with the assumption that cross-correlations between different reservoirs are absent. Note that here any bath-induced renormalization of the transition frequencies is included in $H_S$. Given these rates, we can specify that the above time evolution equations are consistent if $\gamma (\omega _{\alpha \beta })\hbar \beta \ll 1$ and $\gamma (\omega _{\alpha \beta })/\omega _{\alpha \beta }\ll 1$.

Even though Eq. (5) appears as an operator equation, implicit in its derivation is the representation of the density in the eigenstate basis of $H_S$. Making this explicit leads to two sets of equations, one for the diagonal elements (populations) $P_\alpha =\langle \alpha |\rho |\alpha \rangle$ which reads

9 $$\begin{aligned} \dot{P}_\alpha (t) &= \sum _{\beta ,\nu } \gamma _\nu (\omega _{\alpha \beta }) |\mathinner {\langle {\alpha }|}A_\nu (\omega _{\alpha \beta })|\beta \rangle |^2 P_\beta (t) \\ & \quad -\gamma_\nu (\omega _{\alpha \beta }) |\mathinner {\langle {\alpha }|}A_\nu (\omega _{\beta \alpha })|\beta \rangle |^2 P_\alpha (t), \end{aligned}$$

and one for the off-diagonal elements (coherences)

10 $$\begin{aligned} \dot{\rho }_{\alpha \beta }(t) = -\left( i\omega _{\alpha \beta } + \Gamma _{\alpha \beta } \right) \rho _{\alpha \beta }(t), \ \ \alpha \ne \beta \, . \end{aligned}$$

Here, rates are given by

11 $$\begin{aligned} \Gamma _{\alpha \beta } = \frac{\Gamma _{\alpha \alpha }+\Gamma _{\beta \beta }}{2} + \frac{\gamma (0)}{2}\, , \end{aligned}$$

where $$\Gamma _{\alpha \alpha } = \sum _{\beta \ne \alpha , \nu } \gamma _\nu (\omega _{\alpha \beta })$$.

A well-established and very powerful way to describe the effective impact of thermal environments is to model them as quasi-continua of harmonic modes with spectral distribution $J(\omega )$. This modeling is always possible if the fluctuation properties of a reservoir are Gaussian with zero mean which is the natural situation for reservoirs with a macroscopic number of degrees of freedom. The impact of such a reservoir onto a dedicated system is then completely captured by the second order cumulant of the bath force acting onto the system which in turn is determined solely by the spectral density $J(\omega )$ and temperature. Accordingly, the above transition rates can be expressed as

12 $$\begin{aligned} \gamma _\nu (\omega ) = J_\nu (\omega )\left[ \coth (\tfrac{\omega \beta }{2}) - 1 \right] \end{aligned}$$

with the detailed balance property

13 $$\begin{aligned} \gamma _\nu (-\omega ) = J_\nu (\omega )\left[ \coth (\omega \beta /2) + 1 \right] . \end{aligned}$$

The second choice for the dissipators is to model the system-bath interaction as a coupling of each site with individual reservoirs (corresponding to the local dissipation introduced above). This can be done by making explicit the definition of the projection operators over each site $|jl\rangle$, see (2), as a linear combination of the system eigenstates, i.e.,

14 $$\begin{aligned} A_{\nu ,jl} = |jl\rangle \mathinner {\langle {jl}|}A_\nu |jl\rangle \mathinner {\langle {jl}|} = \sum _{n,m} c_{nm, jl}|\alpha _n\rangle \, a_{jl} \mathinner {\langle {\alpha _m}|}\, , \end{aligned}$$

with $a_{jl}=\mathinner {\langle {jl}|}A_\nu |jl\rangle$ and coefficients $c_{nm, jl}=\langle n|jl\rangle \langle jl|m\rangle$ and then using these dissipators for the equations following Eq. (7). Note that this representation of the dissipator in terms of the eigenstate basis avoids an often used but somewhat inconsistent representation in the local site basis. Strictly speaking, this latter representation is only valid if the tunneling terms in the total Hamiltonian (1) are absent, i.e. in the trivial case of non-interacting bases.

In the following, we will define the second choice of dissipators (see Eq. 14) as *Local Dissipators (LD)*, while we will refer to the first choice of dissipators (Eq. 7) as *Global Dissipators (GD)*. Whereas LDs refer to the interaction of a single individual base with the environment, GDs refer to the more global interaction of a set of bases with an external agent, such as the DNA backbone.

Finally, we consider a broadband spectral reservoir distribution of the form

15 $$\begin{aligned} J(\omega ) = \eta \frac{\omega \Omega ^2}{\Omega ^2+\omega ^2} \end{aligned}$$

with a coupling rate $\eta$ and a large cut-off frequency $\Omega$. This type of environmental frequency distribution (also called ohmic-type) describes in the high temperature limit a white noise characteristics of the environment.

### Introducing disorder and correlations in the energetics and dynamics of the system

Correlated fluctuations in local energies of the system are implemented inspired by an approach proposed by Liu et al^23^. However, here we focus on the full quantum dynamics along the chain. First, we introduce a measure of the spatial correlation of the particle dynamics along the chain, defined by

16 $$\begin{aligned} \chi (i)_\sigma ^{d_0} = \frac{<P_0(t)P_i(t)>_t^{d_0, \sigma }}{<P_0(t)P_0(t)>_t^{d_0, \sigma }}\, . \end{aligned}$$

Here, the symbol $<\cdot >_t^{d_0, \sigma }$ refers to the time average for a given choice of parameters $\sigma$ and $d_0$ (be described below), and $P_i(t)$ is the population over time at site *i* of the system investigated. The above measure allows us to track how correlated fluctuations of on-site energies affect the long-range charge propagation, and how such correlated noise might affect long-range coherence.

The noise profile for a given chain is assumed to be constant over the time span of a single run of the model’s dynamics. For each run, a noise profile of degrading correlation strength with distance between sites is generated as follows: First, a value is extracted from a normal distribution with zero mean and variance $\sigma$ to be specified (it represents the strength of the energy fluctuations in the systems). This value is added to the local energy of the top left base of the system, representing its energetic disorder. The energy fluctuation of its neighboring base is then extracted from another Gaussian curve, now averaged around the value found for the first fluctuation and with a variance $\sigma ^{*}$ derived as follows

17 $$\begin{aligned} (\sigma ^*)^2 = 2\sigma ^2\left( 1-C_{ij} \right) \, , \end{aligned}$$

where $C_{ij} = e^{-\frac{|i-j|}{d_0}}$ is the correlation strength between the two bases. Here $d_0$ represents the coherence length of the correlated noise (the longer $d_0$, the higher the correlation of the noise distribution in space, with the limit $\sigma ^* \xrightarrow 0$ for $d_0 \xrightarrow \infty$). Thus, the further away two bases are, the larger the value of $\sigma ^*$, resulting in noise profiles that are increasingly uncorrelated with distance. A more detailed derivation of such a result is given in the supplementary material of Liu et al^23^, including the possibility of accounting for temporal correlations in the energy fluctuations, which is beyond the scope of this paper.

In a previous work, Kubař et al^24^ reported that the average correlation strength between neighboring G sites on a DNA strand is $C^G_{i+1, i} \sim 0.7$, corresponding to a value of $d_0 \sim 2.8$. For this reason, in our investigation, we explore different choices of the parameter $d_0$ ranging from 1 to 5 and $d_0=10$ for neighboring bases. The intensity of the energy fluctuations, modeled by the parameter $\sigma$, is studied under different conditions, ranging from $\sigma = 0.1 \mathrm meV$ to $\sigma = 100 \mathrm meV$, in order to cover both weakly and strongly perturbed regimes with respect to the typical interaction energies of the system under study (see Table 1).

## Results

### Survival of quantum phenomena in DNA-like systems interacting with the environment

To investigate charge dynamics on DNA strands, we use specific parameter sets to mimic three different oligonucleotide sequences: a uniform sequence 5’-GGGGG-3’ (and complementary 5’-CCCCC-3’), a sequence 5’-GCGCG-3’ (complementary 5’-CGCGC-3’), and a sequence 5’-GCACG-3’ (complementary 5’-CGTGC-3’). These short DNA-oligomers are chosen to cover both trivial sequences (poly(dG) - poly(dC) in the present work), which have been already well studied in the literature^25–27^, and more complex ones. The latter were chosen because the resulting dynamics are of potential relevance for epigenetic processes, particularly DNA methylation, which usually occurs at the cytosine in CpG dinucleotides. On the other hand, it has been shown that charge transfer is often particularly facilitated by homopolymers and uniform systems (see Ref.^28^ and references therein). Thus, in addition to a poly-G stretch, a sequence with alternating CGs in both strands was chosen. The cytosines in the CpG context can be methylated by sequence-specific DNA methyltransferases, a process which has experimentally shown interesting interacting features with charge transfer along the DNA, in some contexts even supporting coherent charge transfer^29,30^. For comparison, in the sequence 5’-CGTGC-3’ after a first CpG, the sequence was interrupted by a T and inverted to a GpC. Finally, in the Supplementary Material, we show in Fig. S2 the results for charge dynamics along a sample sequence 5’-TATAT-3’ as a benchmark against C- and G-rich sequences. Even in this scenario, the charge moves from its initial position over the thymine to the next diagonal thymine undergoing a tunneling process through the adjacent adenine base, even in the presence of an external bath.

Corresponding tight-binding parameters are taken according to the work of Hawke^20^ et al. Though in the literature there exist different results (^21,31,32^ and references therein) for the parameters we are using in this model, the ones we chose provide, to our current knowledge, the most comprehensive collection of data needed for our purposes based on Linear Combination of Atomic Orbitals (LCAO) calculations. Even with advanced Density Functional Theory (DFT) techniques, which are supposed to yield the most accurate molecular orbitals descriptions, only relatively short chains can be treated, typically no longer than 3-4 bases.

**Fig. 2:**
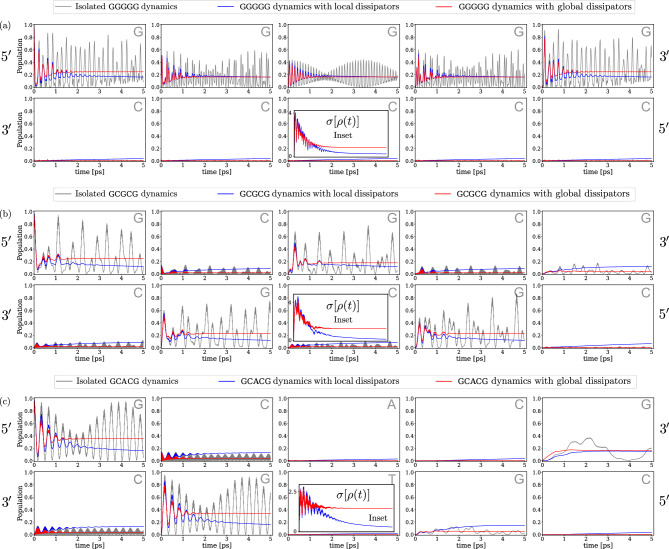
Electron dynamics on DNA segments: Population dynamics in the DNA double-chain of an electron with isolated (grey line) and dissipative time evolution in the presence of a common bath (red line for global dissipation, blue line for local dissipation), for different DNA segments: (**a**) 5’-GGGGG-3’, (**b**) 5’-GCGCG-3’ and (**c**) 5’-GCACG-3’. For all plots, in the bottom-central panel an inset shows the time-evolution of the system coherence during the process.

Figure 2a shows the quantum diffusion of a charge along a ladder consisting of five continuous sites with G (top) - C (bottom) nucleotides, both with and without environmental interactions. Initially, the charge is localized at the leftmost site on the upper strand. As expected, in the isolated case (no environment - grey line) the particle diffuses relatively quickly along the upper G strand with almost no transfer to the complementary C strand though. This is due to the large differences in on-site energies between the G and C sites, which are more than an order of magnitude larger than the energies associated with the overlap of the molecular orbitals of the two bases (see Table 1).

The dynamics resulting from the two different models taking into account the environment interactions, i.e. *GD* dissipation describing the backbone global influence (red in Fig.2a) and *LD* dissipation describing individual interactions of each base with the surrounding environment (blue in Fig.2a) shows some interesting differences: The *GD* partially preserves the coherence of the state even after longer times, whereas the *LD* shows a rapid decay of the coherence of the quantum state. On the other hand, the charge dynamics induced by the *LD* is able to populate sites that are not accessible in both the isolated case and the *GD* scenarios, as shown in Fig. 2a, where the blue line begins to populate sites on the bottom strand that are otherwise never populated by the other two modeled dynamics.

Figure 2b displays a more intriguing behavior of the quantum diffusion along the chain. Here, an alternating top chain 5’-GCGCG-3’ with its complementary sequence on the bottom side (5’-CGCGC-3’) is studied. Apparently, transfer between adjacent Gs is favored compared to the population of intermediate Cs. This is due to the energetically higher-lying Cs compared to the Gs (onsite energies differ by 0.2 eV). This process, also referred to as superexchange^33^, represents a foundational example of particle dynamics governed by quantum (i.e. non-classical) physics within tight-binding models specifically parameterized to reflect the energy topology of DNA. However, since the transfer integral (coupling) G-C is relatively strong, we do not observe a pure G-G superexchange mechanism, but rather a mixed scenario involving weak G-C hybridisation and thus some population transfer to intermediate C sites. A purely superexchange mechanism can be seen instead in Fig. 2c for the 5’-GCACG-3’ (5’-CGTGC-3’) chain, where it is additionally favorable for the charge to tunnel even through the A-T base pair acting as a barrier in the middle of the chain. These data suggest that even in short and relatively simple DNA sequences, the quantum dynamics is prone to an interplay between quantum delocalization and back-scattering (i.e. the reflection of electron waves caused by variations in local energy levels) due to the complex energy landscape along the chain.

A huge difference between the quantum dynamics in the *LD* and *GD* schemes appears along chains of the 5’-GCGCG-3’ and 5’-GCACG-3’ types, as seen in Fig. 2b and c. If reservoirs couple through local operators, we see complete destruction of coherence with a coherence measure $\sigma (t)$ quickly dropping below the value of 0.5. In addition, we can observe population dynamics along the chain that differs substantially from the corresponding isolated time-evolution: While the isolated time-evolution is dominated by tunnelling through neighboring sites, thus populating almost only alternating G sites, *LD* dynamics quickly starts to populate almost equally the entire chain, thus disrupting this structure of the charge distribution. In essence, these results could suggest that thermal reservoirs kill coherent dynamics on a shorter time scale than the one required for the chain to show long-range quantum mobility. The dynamics resulting from a *GD* system-bath coupling scheme presents a very different behavior though. While we are still witnessing a gradual suppression of coherence, both 5’-GCGCG-3’ and 5’-GCACG-3’ sequences achieve higher values of coherence in their dynamics, and their trends suggest that coherence will not collapse to zero for long times. Alongside these results, we can also see major differences in the population dynamics. Indeed, while interferences and revivals along the chain are gradually washed out, only those specific sites that are also populated in the isolated dynamics discussed above get populated. Thus, the features arising from the topography of energy barriers (quantum tunneling) remain basically unchanged.

The main finding of these first modeling steps can be summarized as follows: The different modalities by which the environment couples to DNA-like double-stranded lattices with a complex energy landscape, namely, either non-local *GD* or local *LD*, has a tremendous impact on the predicted charge diffusion process. It is not proven in the literature which coupling scheme is more suitable to mimic the DNA and its environment realistically. In particular, there is no evidence to assume that DNA is predominantly locally coupled to environmental degrees of freedom at the single base level. Our analysis suggests that a more subtle understanding of the interplay between charge mobility along DNA lattices and quantum effects is required for a realistic description, leading to quantitative agreements with first experimental findings that report the existence of coherent ’islands’ of up to 4-5 bases^34–36^.

**Table 1:** TB modeling parameters: energy parameters for modeling the DNA double-strand on the TB lattice. Left (right) sections show the parameters to model the dynamics of a charge sitting on the LUMO (HOMO) orbitals, namely on-site energies for each DNA base (in eV) and intra-chain and inter-chain (last row) transfer integrals for the neighboring DNA bases and base pairs in the charge transfer model (in meV)^20^.

Electron (LUMO orbitals)				Hole (HOMO orbitals)			
On-site energies				On-site energies			
Adenine	Thymine	Guanine	Cytosine	Adenine	Thymine	Guanine	Cytosine
− 4.4 eV	− 4.9 eV	− 4.5 eV	− 4.3 eV	− 8.3 eV	− 9.0 eV	− 8.0 eV	− 8.8 eV

Transfer integrals				Transfer integrals			
DNA bases	Tr. integrals	DNA bases	Tr. integrals	DNA bases	Tr. integrals	DNA bases	Tr. integrals
AA	16	AT	7	AA	− 8	AT	68
AC	− 3	AG	1	AC	68	AG	− 5
TT	− 30	TA	− 7	TT	− 117	TA	26
TC	22	TG	− 17	TC	− 86	TG	28
GG	20	GA	30	GG	− 62	GA	− 79
GT	− 32	GC	43	GT	73	GC	80
CC	− 47	CA	− 12	CC	− 66	CA	5
CT	63	CG	15	CT	− 107	CG	− 1
A-T	− 9	G-C	16	A-T	− 12	G-C	− 12

### Comparing electron and hole dynamics in DNA-like models

The methods we have applied so far can also be used to study the quantum diffusion of holes (lack of an electron in the HOMO band) along DNA-mimicking structures. To do so, we chose a different set of parameters to mimic the behavior of this type of quasi-particles on double-chain sites. This set is reported in Table 1 and is also taken from Hawke et al^20^. In particular, we can compare these results with those simulating the dynamics of an electronic charge along the same chain. Interestingly, the combined electron-hole dynamics may give rise to the formation of excitons which is discussed by Herb et al in^7^.

**Fig. 3:**
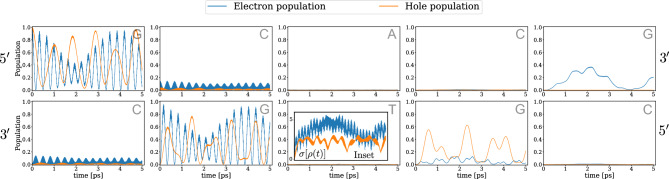
Electron and hole compared dynamics: population dynamics in the 5’-GCACG-3’ double-chain for isolated time evolution and for both electron (blue) and hole (orange) transfer. In the bottom-central panel, an inset shows the time-evolution of the system coherence during the process for both electron and hole.

The results of the simulations are shown in Fig. 3, where for clarity only the results for the 5’-GCACG-3’ sequence are shown, as these are the most relevant. We have already shown above that on this DNA segment the electron is able to tunnel through the DNA sites to populate the guanine site on the right side of the chain. The hole dynamics, however, shows different tunneling characteristics. Namely, being able to populate the two guanine sites on the left side of the chain in a similar way to the electron dynamics, its behavior changes dramatically on the right side, where the hole can only occupy the guanine site on the lower strand.

How electrons and holes populate the right-hand side of this chain can potentially be a strong source of resistance to exciton annihilation, i.e. the release of the energy contained in the initially excited electron, which then falls back into its original equilibrium molecular orbital. In fact, the probability that an electron will relax back into the orbital from which it was excited depends strongly on the overlap of wavefunctions characterizing the electron and hole states at any point in time during the dynamics. Thus, if they are “trapped” far away from each other, the probability for excitonic annihilation is greatly reduced. As a possible consequence, long-lived excitation phenomena as observed in different experimental setups^37–39^ could be explained in this way. These results suggest that investigating more thoroughly electron-hole relaxation could be critical to finally being able to link yet not completely understood experimental results with theoretical modelling.

**Fig. 4:**
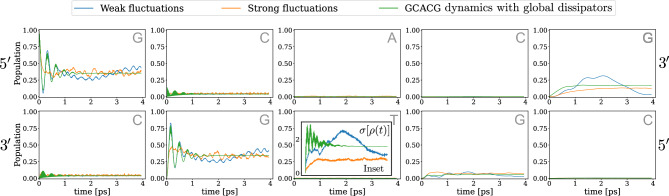
Electron dynamics with energy disorder: Population dynamics in the 5’-GCACG-3’ double-chain for static disorder and global Lindblad dissipation time evolution in electronic transfer. In the bottom-central panel, an inset shows the time-evolution of the system coherence during the process for all the dynamics plotted.

**Fig. 5:**
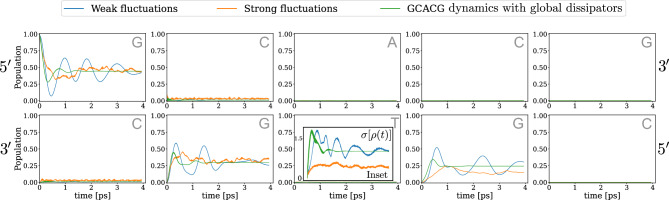
Hole dynamics with energy disorder: population dynamics in the 5’-GCACG-3’ double-chain for static disorder and global Lindblad dissipation time evolution in hole transfer. In the bottom-central panel, an inset shows the time-evolution of the system coherence during the process for all the dynamics plotted.

### Investigating the impact of parametric energy disorder on the dynamics of DNA-like systems

Next, we consider another source of decoherence, the tendency for charge localization that can arise from disorder in the local energy parameter distribution of DNA bases along the double strand, as opposed to the proximity-correlated energy parameter disorder discussed in the next section. Under the influence of mutating external force fields, generated by ions and charges moving in the surroundings of the DNA in a cell, the energies characterizing the DNA structure itself can undergo local fluctuations. As suggested by different publications^40–42^, dynamic fluctuations in the energetics of the system can strongly affect how a particle propagates along the DNA chain, and this effect can also differ between electron and hole dynamics. We describe this by running several different dynamical simulations over the same DNA sequence, 5’-GCACG-3’, where the site energies $\epsilon _{j, l}$ in Eq. (2) are replaced by $\epsilon _{i, l}+\xi _{i, l}$ with $\xi _{i, l}$ being a random variable sampled from a Gaussian distribution with zero mean and variance to be set as an external parameter. We use two different values for the variance, the weaker one resembling energetic fluctuations typical of body temperature processes (about 27 meV), and the second, inspired by Gutiérrez et al.^42^ (360 meV), aiming to include even more sources of disorder, such as interactions with ions and solvent degrees of freedom.

In Figs. 4 and 5 we show the dynamics for an electron and a hole, respectively, both on the same DNA sequence, plotted together with their dynamics when in contact with a thermal bath modeled by global Lindblad operators. As for the plots above, the gross coherence dynamics for these simulations can be found in the bottom right panel’s inset. It is interesting to note that the influence of static disorder on the dynamics of the particles is very similar to that of the global Lindblad operators. This is due to the fact that both sources of decoherence have the tendency to preserve the structure of eigenstates of the system, thus both mimicking low frequency fluctuations.

As a result, it seems evident that the main considerations we made in the previous Section about the relationship between electron and hole dynamics along the 5’-GCACG-3’ strand hold true even in the presence of static disorder, both for small and large variations in the on-site energies, since this electron-hole separation effect appears stable under this noise source.

### Impact of proximity-correlated parametric energy disorder on the dynamics of DNA-like systems

Finally, we turn our attention to the effect of proximity-correlated fluctuations, as opposed to the uncorrelated ones presented in the previous Section. Proximity-correlated fluctuations in the local energy parameters of the system are implemented inspired by an approach proposed by Liu et al.^23^ on the 5’-GGGGG-3’ strand. The reason for choosing this strand is to demonstrate the effect of correlated low-frequency noise on charge dynamics in a system with an already structurally high degree of charge mobility. The measure introduced in the Methods above allows us to track how correlated fluctuations of on-site energies affect the long-range charge propagation, and how such correlated noise may be beneficial/detrimental to support long-range coherence.

**Fig. 6:**
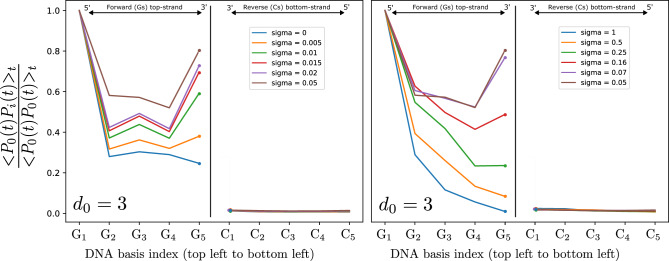
Correlations within electron dynamics under noisy conditions: correlation measure for the time-averaged particle dynamics along each site (x-axis) of a double-stranded 5’-GGGGG-3’ chain, for different values of the noise level ($\sigma$ parameter). The numerical labeling of the basis goes left to right for both strands (G$_1$ top left, C$_1$ bottom left).

**Fig. 7:**
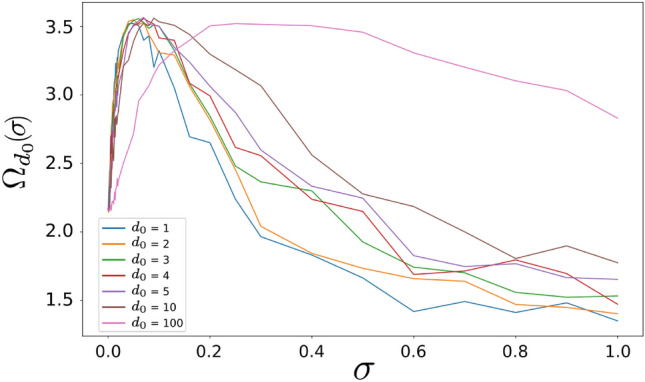
Estimated total effect of noise on electronic dynamical correlations: total correlation along a chain (sum over each site) for increasing $\sigma$ values (x-axis), for different correlation lengths. Smaller values of $\sigma$ are evaluated more finely (logarithmic selection of values).

Figure 6 shows, for a fixed parameter $d_0 = 3$ as a reference, the time-averaged correlation between the charge dynamics at each site of the DNA strand with respect to the dynamics at site $G_0$. The two plots show the same scenario for different choices of sigma. The left plot displays the degree of dynamical correlation of each site of the double chain for increasing values of the parameter $\sigma$, from 0 to 0.05. The right plot instead starts from the results for $\sigma = 0.05$ and increases this value up to $\sigma = 1$. First, we can see that the correlation for the sites on the opposite strand of Cs is always zero. This reflects the fact that due to the energy topology the quantum particle only delocalizes along the upper G-strand and never travels to the opposite chain. More interestingly, however, the data demonstrate that correlations along the G strand increase significantly with growing noise level, up to the value of $\sigma = 0.05$ (left plot in Fig. 6), and only for even larger values begin to decrease, as would normally be expected from a measure of correlation versus noise level (right plot in Fig. 6). The same can be observed for different values of $d_0$.

To better investigate this aspect and to compare scenarios for different values of $d_0$, we considered the following quantity

18 $$\begin{aligned} \Omega (\sigma )^{d_0} = \sum _{i}\chi (i)_\sigma ^{d_0} \end{aligned}$$

which is plotted in Fig. 7. It is trivial to note that for $\sigma = 0$ all curves converge to a fixed value, since for zero fluctuations the contribution of the $d_0$ parameter is nullified. Moreover, along the rest of the curves, for larger values of $d_0$, the overall correlation $\Omega$ is generally higher for each value of $\sigma$ considered, reflecting the fact that for longer correlation lengths one expects a higher degree of correlation between the dynamics at different sites.

More importantly, however, these results illustrate that, for any value of $d_0$, the maximum degree of correlation *cannot* be found for $\sigma = 0$, as one would expect naively from the zero noise condition, but for a finite value of $\sigma$. Its specific value depends on the correlation length of the system under study. Hence, this analysis suggests that the presence of correlated static perturbations along the DNA does not necessarily disrupt coherent propagation but may even *support* it to some extent, in line with previous experimental findings^34–36^.

## Discussion

In this study, we modelled charge diffusion in two-dimensional DNA-inspired lattices with the main focus on the impact of various noise mechanisms. These ranged from thermal interactions with environmental degrees of freedom to global interactions with the backbone, as well as proximity-correlated parametric energy disorder, i.e. disorder arising from vibrations or other sources that propagate along the double chain inducing long-range correlations over several bases. The motivation for this study was triggered on the one hand by recent technical developments exploiting long-range charge transfer through DNA, and on the other hand by potential biological implications of charge (de)localization in DNA for the regulation of DNA repair and replication, in particular epigenetic mechanisms of gene expression including DNA modification or binding of transcription factors (^8^ and references therein).

The lattice model used here includes intra- and inter-strand site couplings and initial states prepared on one of the sites. For DNA lattices, parametrized according to atomistic simulations, in the absence of noise the energetic profile along complementary chains promotes fast population transfer for spatially separated sites due to long-range tunneling related to long-range coherences due to proper $\pi$-stacking of bases.

Weak coupling to thermal reservoirs substantially modifies this picture, however, with a strong dependence on the details of the coupling mechanism (local versus global). This adds to the high sensitivity with respect to the specific sequence of nucleobases which determines the overall energetic landscape for charge transfer. While oscillatory patterns in the population dynamics die out rather quickly already for weak decoherence, interestingly, intra-strand coherences survive on relatively long times scales. For global quantum noise, coherences are present on much longer time scales, which may be of use in setups where molecular structures are placed in cavities.

Electron and hole diffusion happen to be quite different in DNA-like lattices, which can lead to complex exciton transfer in bare and weakly dissipative chains, as further investigated in Herb et al.^7^ for isolated dynamics. This phenomenon appears to survive in the presence of both Lindblad-like dissipation as well as for disorder noise and may stimulate further investigations to explore possible biological relevance.

The impact of disorder on on-site energies also reveals a rich and interesting behavior: local parametric energy disorder, at both low and high intensities, exhibits dynamics similar to those induced by global dissipation, which is reasonably expected. In particular, when considering such disorder at room temperature energies, simulations suggest that coherences along the chain are not suppressed on ultrafast timescales, but rather tend to have a relatively long-lived behavior. Proximity-correlated parametric energy disorder over several nucleobases reveals a somewhat counterintuitive but even more intriguing scenario, in that a certain degree of disorder even supports fast and coherent charge propagation along double-stranded DNA.

These results, based on the simple ladder model for DNA, indicate the potential importance of diagonal 5’-5’ and 3’-3’ interactions. Having identified the effects of different classes of noise, we are now in a position to provide a more refined analysis based on the extended ladder model (see^20,21,43^) in future work. Another interesting direction is tailored DNA sequences, where DNA does not appear in its native form as a carrier of genetic information, but rather as a tool to control quantum states in designed aggregates that are molecular or semiconductor-like in nature. Another line of research could open up a new interdisciplinary field at the interface of physics, chemistry and genetics (as suggested in^8^): by combining experimental findings on long-range charge transfer^30^ with quantum mechanical simulations of the type presented here, we may on the one hand be able to elucidate the potential role of such processes in efficiently screening DNA sequences for integrity under ambient conditions. On the other hand, advanced models based on the present findings might also shed further light into epigenetic mechanisms like DNA methylation, which is known to affect fast charge transfer and conductance along DNA. However, a detailed understanding of this and analogous phenomena remains to be developed.

## Supplementary Information

Supplementary Figures.

## Data Availability

The data that supports the findings of this study are available within the article and the software suite used to generate our findings is available at https://zenodo.org/doi/10.5281/zenodo.14450293^44^] or http://dx.doi.org/10.6084/M9.FIGSHARE.28025372^45^].
