# Supplementary material for: Effects of environmental noise on quantum charge diffusion in DNA sequences
Source: Sci Rep. 2025 May 20;15:17543. doi: 10.1038/s41598-025-02819-w (PMC12092660; doi:10.1038/s41598-025-02819-w)
Supplement: Supplementary file 1 — Supplementary Figures. [file 41598_2025_2819_MOESM1_ESM.pdf]

Supplementary Material: Effects of environmental noise on quantum charge diffusion in DNA sequences

Mirko Rossini[1,2,*], Ole Ammerpohl[3], Reiner Siebert[2,3], and Joachim Ankerhold[1,2]

[1]Ulm University, Institute for Complex Quantum Systems, Ulm, 89069, Germany
[2]Center for Integrated Quantum Science and Technology (IQST), Germany
[3]Ulm University and Ulm University Medical Center, Institute of Human Genetics, Ulm, 89069, Germany
[*]mirko.rossini@uni-ulm.de

An example of eigenenergies distribution for an increasingly long double-stranded TB model

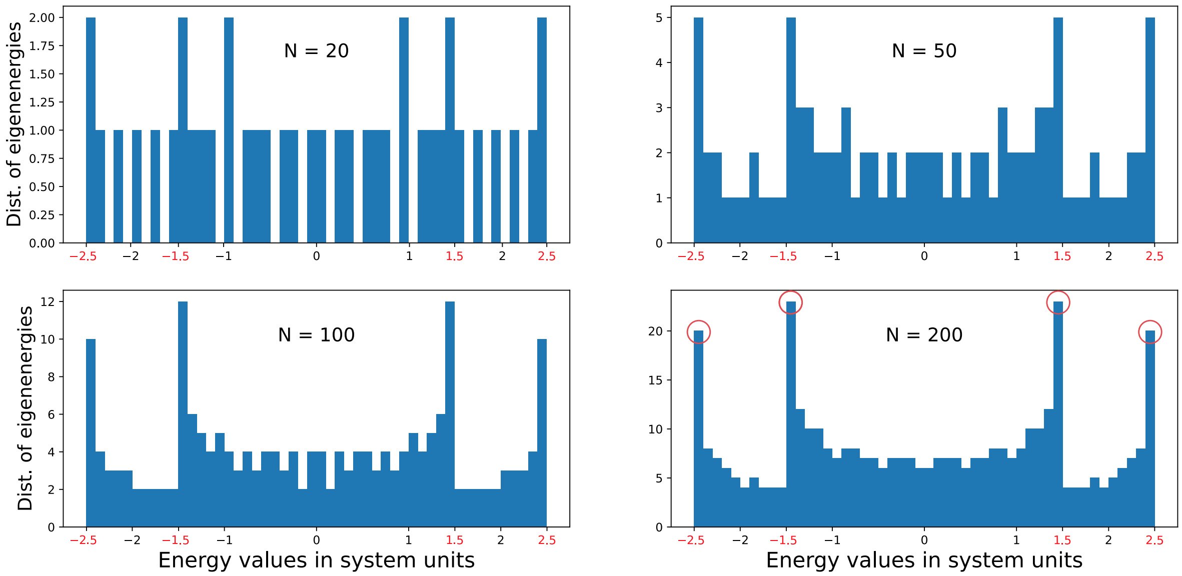

Figure 1. : Figure S1

The figure above shows the distribution of eigenenergies (in units of w) of the system described by the Tight-Binding lattice in Fig.1 in the manuscript, as a function of the total number of sites N. As can be shown analytically, all the values range between -2.5 and 2.5, with peaks (highlighted with red circles) corresponding to energetic regions with a high density of states.

Benchmarking example for sequences without G-C pairs

We provide in the following the charge dynamics simulation for a sequence, 5'-TATAT-3', which is free of GC base pairs as a benchmark. Fig. S2 shows the particle dynamics and the coherence evolution for the three scenarios with no bath, local dephasing and global dephasing. Even in this scenario, the charge moves from its initial position over the thymine to the next diagonal thymine undergoing a tunneling process through the adjacent adenine base, even in the presence of an external bath.

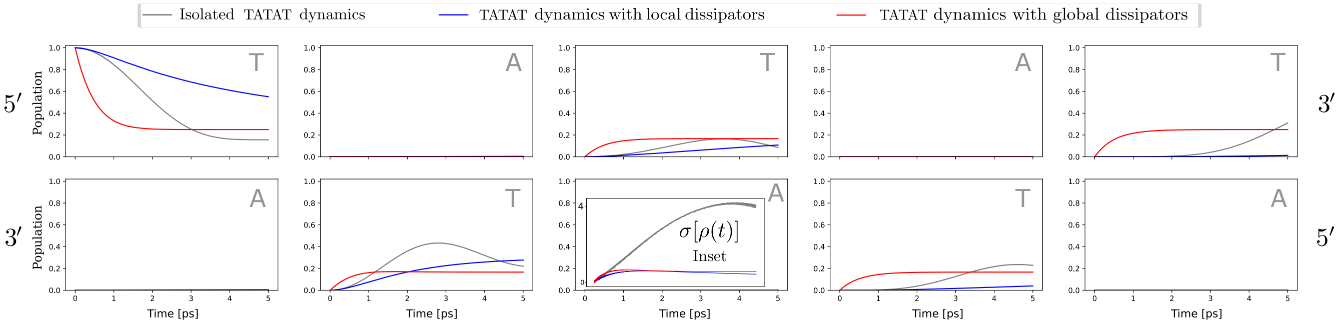

Figure 2. : Figure S2
